# Transcription factor BACH1 in cancer: roles, mechanisms, and prospects for targeted therapy

**DOI:** 10.1186/s40364-024-00570-4

**Published:** 2024-02-07

**Authors:** Dian Hu, Zerui Zhang, Xiangyuan Luo, Siwen Li, Junqing Jiang, Jiaqian Zhang, Zhangfan Wu, Yijun Wang, Mengyu Sun, Xiaoping Chen, Bixiang Zhang, Xiao Xu, Shuai Wang, Shengjun Xu, Yufei Wang, Wenjie Huang, Limin Xia

**Affiliations:** 1 Department of Gastroenterology, Institute of Liver and Gastrointestinal Diseases, Hubei Key Laboratory of Hepato-Pancreato-Biliary Diseases, Tongji Hospital of Tongji Medical College, Huazhong University of Science and Technology, Wuhan, 430030 Hubei Province China; 2 Hubei Key Laboratory of Hepato-Pancreato-Biliary Diseases; Hepatic Surgery Center, Tongji Hospital, Tongji Medical College, Huazhong University of Science and Technology; Clinical Medicine Research Center for Hepatic Surgery of Hubei Province; Key Laboratory of Organ Transplantation, Ministry of Education and Ministry of Public Health, Wuhan, 430030 Hubei China; 3 Key Laboratory of Integrated Oncology and Intelligent Medicine of Zhejiang Province, Department of Hepatobiliary and Pancreatic Surgery, Affiliated Hangzhou First People’s Hospital, Zhejiang University School of Medicine, Hangzhou, 310006 China; 4 Key Laboratory of Integrated Oncology and Intelligent Medicine of Zhejiang Province, Department of Hepatobiliary and Pancreatic Surgery, Affiliated Hangzhou First People’s Hospital, Westlake university school of medicine, Hangzhou, 310006 China

**Keywords:** BACH1, Cancer, Immunity, Targeted therapy, Prognostic biomarker

## Abstract

Transcription factor BTB domain and CNC homology 1 (BACH1) belongs to the Cap ‘n’ Collar and basic region Leucine Zipper (CNC-bZIP) family. BACH1 is widely expressed in mammalian tissues, where it regulates epigenetic modifications, heme homeostasis, and oxidative stress. Additionally, it is involved in immune system development. More importantly, BACH1 is highly expressed in and plays a key role in numerous malignant tumors, affecting cellular metabolism, tumor invasion and metastasis, proliferation, different cell death pathways, drug resistance, and the tumor microenvironment. However, few articles systematically summarized the roles of BACH1 in cancer. This review aims to highlight the research status of BACH1 in malignant tumor behaviors, and summarize its role in immune regulation in cancer. Moreover, this review focuses on the potential of BACH1 as a novel therapeutic target and prognostic biomarker. Notably, the mechanisms underlying the roles of BACH1 in ferroptosis, oxidative stress and tumor microenvironment remain to be explored. BACH1 has a dual impact on cancer, which affects the accuracy and efficiency of targeted drug delivery. Finally, the promising directions of future BACH1 research are prospected. A systematical and clear understanding of BACH1 would undoubtedly take us one step closer to facilitating its translation from basic research into the clinic.

## Background

Transcription factor BACH1 belongs to the CNC-bZIP family and contains 736 amino acids [1, 2]. BACH1 was initially identified as a heterodimerization partner of MafK, which was discovered through a yeast two-hybrid screen [2]. BACH1 is expressed widely and plays an important role in coordinating the transcriptional activation and inhibition of MafK [2]. *Not*I restriction mapping and YAC contig mapping demonstrated that the BACH1 gene is present at 21q22.1, between *Not*I sites LA329 (D21S338) and LL60 (D21S389), approximately 400 kb away from LA329 [1, 3]. The mouse homolog shares 80.3% sequence similarity with human BACH1 [1]. In the last thirty years, the roles of BACH1 have been extensively studied. BACH1 regulates heme homeostasis and effectively mediates oxidative stress, thus playing an essential role in inflammatory diseases and cancer [4–8]. In addition, BACH1 also exerts essential effects on various malignant biological behaviors of tumors [9–11]. For example, results from our laboratory and others have shown that BACH1 promotes the metastasis of hepatocellular carcinoma, making it a promising novel biomarker predicting a poor prognosis [12].

This review dissects the molecular mechanisms underlying the roles of BACH1 in epigenetic modifications, heme homeostasis, and oxidative stress, with a focus on the hallmarks of cancer, including tumor cell metabolism, invasion and metastasis, proliferation, different cell death ways, drug resistance and tumor immunity. Finally, promising therapeutic strategies are presented by summarizing and analyzing the potential clinical roles of BACH1. At the same time, the current research status of BACH1 as a prognostic biomarker is discussed. BACH1 is expected to become an effective novel target for tumor therapy.

## Structure of BACH1

**Fig. 1:**
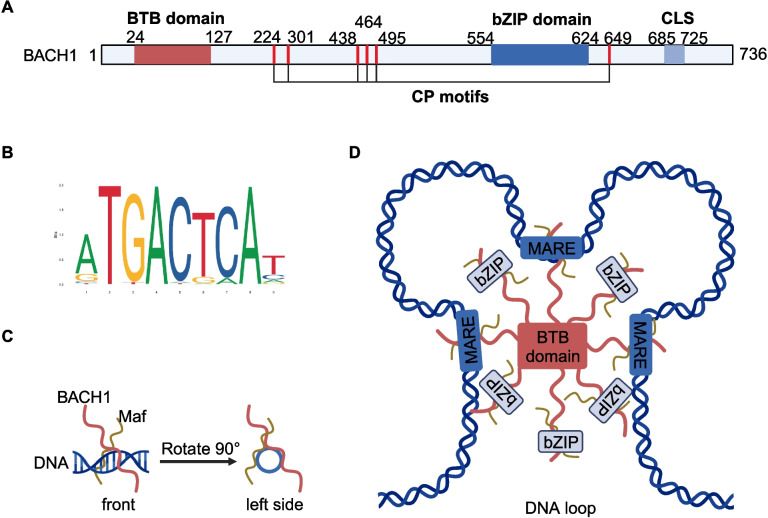
Domain structure and binding profile of human BACH1 transcription factor. **A** Domain structure of BACH1. **B** Binding profile of human BACH1 transcription factor. **C** The interaction between BACH1 and small Maf protein in the presence of DNA via the bZIP domain. **D** A DNA loop is developed through multimeric interactions via the BTB domain

BACH1 contains four functional regions, including a Broad-complex, Tramtrack, and Bric-à-brac (BTB) domain, six cysteine–proline (CP) motifs, bZIP domain, and cytoplasmic localization signal (CLS) (Fig. 1A, B). The N-terminal BTB domain is also commonly called the pox virus and zinc finger (POZ) domain and often appears in the transcription factors with the bZIP domain. The BTB domain interacts with non-BTB proteins and binds to the chromatin structure, an essential step for regulating gene transcription [13]. BACH1 must constitute dimers or oligomers to recognize particular sequences of target genes, and the N-hook motif plays a critical role in BACH1 dimerization [14]. As shown in Fig. 1C, D, the BTB domain of BACH1 regulates homodimerization in vitro, which enables BACH1 to interact with Maf protein and develop a divalent DNA-binding complex that induces a DNA loop [15]. The C-terminal bZIP domain comprises highly conserved fundamental regions, crucial for DNA interactions, as well as less conserved short amphipathic leucine zipper domains that develop a dimeric coiled-coil structure. Furthermore, they interact with other bZIP monomers via bZIP domains [16, 17]. The transcription factors with the bZIP domain dimerize around DNA via leucine zipper interactions, and each monomer’s basic regions combine similar sequences on the corresponding strands of DNA. The core sequences of DNA binding sites at which bZIP monomers develop dimeric interactions are named TPA response elements (TREs) or cAMP response elements (CREs) [18]. The dimers bind TREs or CREs to develop the palindromic sequences TGA(G/C)TCA and TGACGTCA [19]. For example, the bZIP domain mediates the heterodimerization of BACH1 and small Maf proteins around DNA, due to which the heterodimers reduce the expression of many redox homeostasis genes and bind the Maf recognition elements (MAREs) in the gene promoters. In addition, heme can directly bind to the six CP motifs of BACH1, leading to its inactivation, nuclear export, ubiquitination and degradation by hoil-1 [20–22]. This mechanism adjusts the intracellular heme levels. The CLS is a highly conserved structure in BACH1 that leads to its cytoplasmic accumulation, which depends on the nuclear exporter chromosome maintenance 1 (CRM1). The phosphorylation of BACH1 at tyrosine residues by antioxidants and cadmium induces the nuclear export of BACH1, playing a role in the activation of CLS by the extracellular signal-related kinase (ERK) [23, 24]. Similarly, inorganic arsenic modulates the intracellular localization of BACH1, but the details of the underlying mechanism still need to be confirmed [25].

## Epigenetic modifications

Epigenetic modifications affect gene expression by altering DNA and the bound proteins that constitute chromatin through chemical modifications [26]. Numerous studies demonstrate that BACH1 mediates the expression of target genes by inducing DNA methylation and histone modifications. A CpG island methylator phenotype (CIMP) refers to DNA hypermethylation of CpG islands in the promoters of tumor suppressor genes and DNA repair genes, which is a common biological phenomenon and plays a crucial role in chromosomal instability in carcinogenesis, as well as having a high correlation with tumor prognosis [27]. In BRAF (V600E) mutant skin and colon cancer, a complex comprising BACH1 and MAFG is formed, which is promoted by B-Raf proto-oncogene variant BRAF (V600E). The complex recruits DNMT3B and chromatin helicase DNA-binding protein-8 (a chromatin remodeling factor) to the promoters of the mismatch repair gene MLH1 and other target tumor repressor genes containing CIMP. In turn, the hypermethylation of promoters is induced to silence gene expression, thus promoting cancer progression [28, 29]. In addition, p53^R175H^ recruits LSD2, a histone H3K4me1/2 demethylase, and binds to BACH1, thereby forming a p53^R175H^-BACH1-LSD2 complex, which mediates the selective regulation of BACH1 targets by affecting the methylation status of target genomic proteins [30]. BACH1 recruits PRC2 and directly interacts with subunit EZH2 to facilitate the trimethylation of lysine 27 in histone 3 (H3K27me3), thereby silencing the downstream mesendodermal genes such as TBXT, GATA6, and MSX2 [31]. BACH1 recruits NANOG and the MLL/SET1 complex to the chromatin loop. It maintains the trimethylated state of H3K4 and the activity of enhancer-related promoters, which induces the expression of stemness-associated genes in mESCs, such as NANOG, ZFP42, and LIF [32]. In addition to histone methylation, BACH1 competes with β-catenin for complex formation with transcription factor 4 (TCF4). It recruits histone deacetylase 1 (HDAC1) to the promoters of TCF4-targeted genes, thereby inhibiting the expression of VEGF and IL-8, which depends on the BTB domain [33, 34]. In MEFs, BACH1 forms protein complexes with p53, N-CoR, and HDAC1 to inhibit the activation of p53 and is recruited to the promoters of p53 target genes [35, 36]. However, the concrete molecular mechanisms remain unclear and merit further research. The BACH1/Maf heterodimer recruits the complex consisting of NuRD, SIN3A, and SWI/SNF to the locus control region, thereby inhibiting the transcription of the β-globin gene by remodeling chromatin through histone deacetylation [37].

## Heme homeostasis

**Fig. 2:**
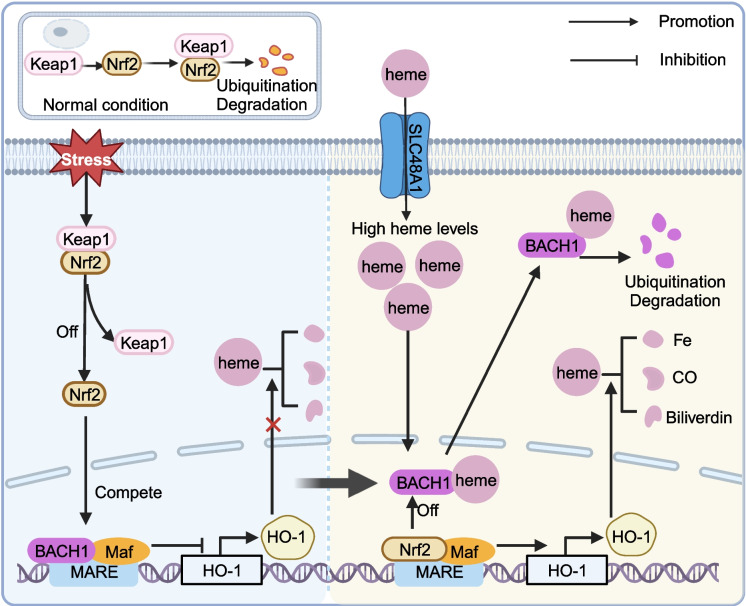
BACH1 plays a vital role in heme homeostasis. BACH1 regulates heme homeostasis by controlling the transcription of the HO-1 gene. The interaction between BACH1 and Nrf2 is also involved in the regulation of heme levels

Heme is a common and critical cofactor involved in various cellular processes [38]. However, free heme that is not bound to proteins can damage cells and tissues by inducing oxidative stress, which underscores the importance of tightly regulating heme levels. Heme oxygenase-1 (HO-1) is a rate-limiting enzyme for heme synthesis that also facilitates its degradation into iron, carbon monoxide, and biliverdin. Typically, BACH1 generates a complex with small Maf proteins to bind to the enhancer of the HO-1 gene and represses its expression [4]. Deficiency of BACH1 disinhibits HO-1 expression, thereby decreasing heme degradation [4]. The regulatory effect of BACH1 on HO-1 is also controlled by nuclear factor erythroid 2-related factor 2 (Nrf2). Under chemical or oxidative stress, Keap1-mediated Nrf2 ubiquitination and proteasomal degradation are inhibited [39–41]. The free Nrf2 is the transported from the cytoplasm to the nucleus, where it competes with BACH1 for small Maf proteins as an activating transcription factor in MAREs and transactivates HO-1 expression (Fig. 2) [4, 42, 43]. At the same time, BACH1 accumulation is induced by the accumulation of free Nrf2 [44]. Conversely, heme-binding BACH1 is exported from the nucleus and degraded by ubiquitination [43, 45].

Accordingly, BACH1 is sensitive to heme levels inside the cell, so that its transcription function and protein levels are mediated by heme. Increasing heme levels abolish the suppression of HO-1 by BACH1/MafK, causing a shift toward Nrf2 activation by repressing the DNA-binding activity of BACH1 [4, 20]. It is reported that heme also binds to CP motifs, thereby boosting the nuclear export of BACH1 and exposing it to ubiquitination by Crm1, which has been demonstrated in diverse cell lines [4, 22, 46]. Conversely, lower heme levels induced by a heme inhibitor (succinylacetone) facilitate the nuclear accumulation of BACH1 in murine embryonic fibroblasts [22]. Overall, the BACH1/HO-1/heme pathway maintains heme homeostasis via a negative feedback loop.

## Oxidative stress

Oxidative stress increases the intracellular levels of reactive oxygen species (ROS), which are generally cytotoxic and can also have an oncogenic effect [47]. BACH1 acts as a dual transcription factor that directly or indirectly regulates oxidative stress under physiological and pathological conditions. Glutathione (GSH) mitigates ROS-induced tissue damage, and cystic fibrosis transmembrane conductance regulator (CFTR) is one of the cellular efflux pumps that export GSH to reduce oxidative stress [48]. Under physiological conditions, BACH1 directly suppresses the expression of the CFTR gene by binding to its -44kb antioxidant response element in complex with MafK [49]. Conversely, nuclear translocation of BACH1/Nrf2 under oxidative stress promotes CFTR expression by competitively binding to MafK [49]. At 20% ambient oxygen, BACH1 also activates CFTR expression by binding to other cis-regulatory elements [50]. Thus, the regulation of CFTR by BACH1 in response to environmental signaling is bidirectional. As a core regulator, the upstream regulation for BACH1 was widely reported in oxidative stress. The genetic ablation of BACH1 upregulates the expression of HO-1 and PGC-1α, thus reducing the production of ROS [51]. In addition, the BACH1/Nrf2 balance is mediated by signal stimulation. Following induction by the receptor activator of nuclear factor-κB ligand (RANKL), BACH1 is transported into the nucleus, thereby suppressing the induction of Nrf2 and facilitating ROS pathways [52]. Following induction by lipopolysaccharide (LPS), BACH1 regulates inflammation and oxidative stress in acute injury, which is associated with Nrf2/HO-1 signaling [53, 54]. Approximately 30% of human lung cancers gain mutations affecting Keap1 or NFE2L2 (encoding Nrf2), which lead to the stabilization of Nrf2, influence the nuclear transport of BACH1/Nrf2, and upregulate the expression of HO-1, thereby combating oxidative stress [22].

## Immune system

BACH1 plays an essential role in the innate and adaptive immune system, especially mediating the development of B lymphocytes and differentiation of macrophages. Firstly, BACH1 expression is particularly promoted in mature lymphoid lineage cells. C/EBPβ (encoded by the Cebpb gene) is a critical transcription factor in differentiating myeloid lineage cells [55]. BACH1 directly suppresses the expression of myeloid lineage-related genes (e.g. Cebpb), thereby suppressing the differentiation of common lymphoid progenitors (CLPs) into myeloid lineage cells [56]. In addition, it is reported that both BACH1- and BACH2-deficient CLPs almost completely lack B cell differentiation in vitro. Similarly, combined BACH1- and BACH2-deficient mice have a decreased abundance of pro-B cells, which was not observed in cases of individual BACH1 or BACH2 deficiency [56]. This means that BACH1 has redundant functions during lymphoid development [56]. Early B cell factor 1 (Ebf1) promotes B lymphocyte lineage differentiation. In both BACH1- and BACH2-deficient CLPs, expression of Ebf1 is inhibited, while sufficient expression of Ebf1 rescues the normal differentiation to B lymphocytes. This suggests that BACH1 indirectly promotes the expression of the Ebf1 gene, thereby inducing pro-B lymphocyte development [56]. Overall, BACH1 makes a difference in the early stages of B cell development and consolidates early B cell features. As indispensable transcription factors in B lymphocytes, BACH1 and BACH2 are negatively modulated by miR-148a, suppressing B lymphocyte maturation and homeostasis together [56–58].

In addition, BACH1 plays a vital role in differentiating red pulp and bone marrow macrophages by controlling heme homeostasis [59]. BACH1 directly or indirectly inhibits Spic expression in monocytes and blocks their differentiation into red pulp and bone marrow macrophages, which is abrogated by heme. At the same time, the heme-mediated upregulation of HO-1 by BACH1 reduces cytotoxicity in red pulp and bone marrow macrophages. The increased abundance of red pulp and bone marrow macrophages greatly enhance red blood cell phagocytosis and lower heme levels. The BACH1/HO-1 pathway also promotes engulfment and the anti-inflammatory process of macrophages during efferocytosis by regulating the engulfment of low-heme apoptotic thymocytes and high-heme red blood cells [60]. Secondly, the BACH1/HO-1 pathway regulates the differentiation of inflammatory macrophages. BACH1 represses the differentiation of M2 macrophages, and one of the underlying mechanisms is probably the inhibition of HO-1 in macrophages by BACH1 [59]. In peritoneal macrophages, BACH1 suppresses the expression of genes involved in their polarization toward the M2 phenotypes, containing those encoding arginase 1, FIZZ1, CD206, and YM1 [61]. Thus, BACH1 controls diverse differentiation events in the macrophage lineage, but the precise mechanisms still need to be better understood and require further research. BACH1 also influences immunomodulatory activities and macrophage reprogramming by cellular bioenergetics. BACH1-deficient macrophages exhibit a mitochondrial metabolic shift, which presents as an increase in glycolysis and a reduction in oxidative phosphorylation (OXPHOS). In addition, BACH1 deficiency in macrophages promotes NLRP3 inflammasome activation by altering iNOS and COX-2 expression upon LPS stimulation [62]. Taken together, the available studies demonstrate that BACH1 has vital functions in the development, differentiation, and functioning of immune cells.

## BACH1 in cancer

BACH1 is known as an essential transcriptional factor that regulates physiological processes. Thus, it is unsurprising that the dysregulation of BACH1 contributes to severe pathological changes, including tumorigenesis. Numerous studies have shown that BACH1 is highly expressed in multiple tumors and boosts their malignant biological behaviors. The following sections will elaborate on the roles of BACH1 in metabolism, invasion, metastasis, proliferation, and different cell death pathways of cancer cells. In addition, we discuss the potential roles of BACH1 in drug resistance and tumor immunity.

### Metabolism

#### Mitochondrial metabolism and aerobic glycolysis

**Fig. 3:**
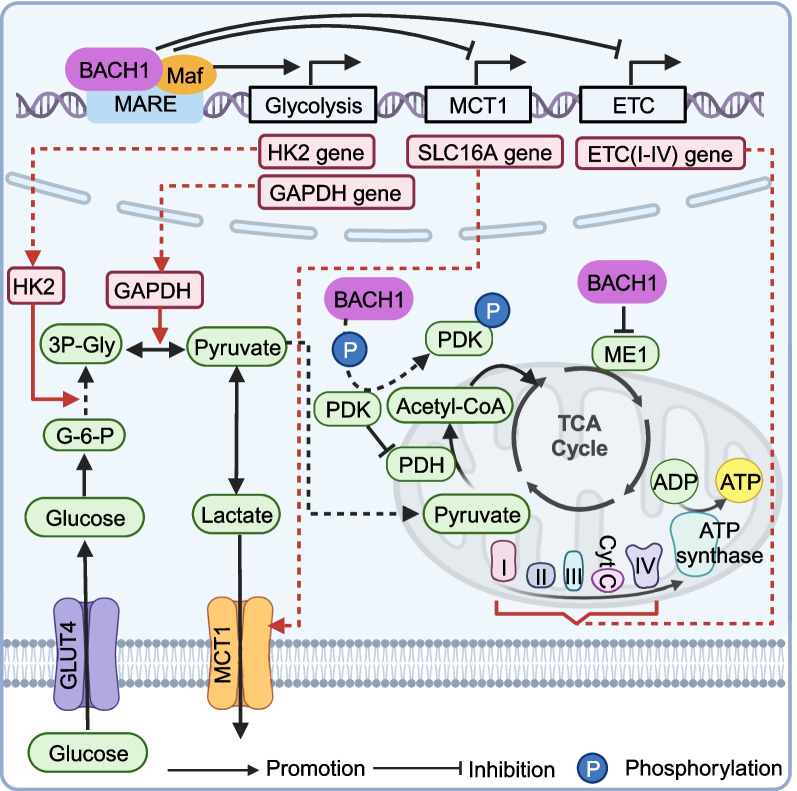
BACH1 affects cancer-cell metabolism thereby promoting tumor progression. BACH1 affects mitochondrial metabolism, aerobic glycolysis, and lactate metabolism in cancer cells by transcriptional regulation

Cells typically gain energy through glycolysis in the cytoplasm, followed by mitochondrial OXPHOS with oxygen. However, cancer cells preferentially employ cytoplasmic glycolysis despite having sufficient oxygen. The phenomenon is identified as the “Warburg effect” or “aerobic glycolysis” [63, 64]. Notably, BACH1 regulates aerobic glycolysis to promote tumor growth, progression, and metastasis in breast cancer, lung cancer, hepatocellular cancer, and glioma by controlling the expression of genes encoding the mitochondrial electron transport chain (ETC) and glycolytic enzymes (Fig. 3). In breast cancer, BACH1 inhibits the expression of ETC complex (I-IV) genes and the activity of pyruvate dehydrogenase (PDH) via pyruvate dehydrogenase kinase (PDK), thereby decreasing mitochondrial metabolism (Fig. 3). BACH1 directly inhibits the mitochondrial ETC gene expression. Bioinformatics analyses support a negative association between the expression of the ETC gene and BACH1 expression in human breast cancer cells [10]. At the same time, deficiency of BACH1 stimulates the expression of the ETC gene, promotes mitochondrial respiration, and improves glucose utilization via the tricarboxylic acid (TCA) cycle [10]. Notably, the mitochondrial ECT complex is usually coupled to the TCA cycle. In addition, BACH1 increases the phosphorylation of both PDK and PDH on S293 but does not influence the PDH level to promote pyruvate decarboxylation. In lung cancer cells, BACH1 promotes the expression of glycolytic enzymes, such as hexokinase2 (HK2) and glyceraldehyde-3-phosphate dehydrogenase (GAPDH), to support aerobic glycolysis, which is accomplished by binding to the promoter regions of their encoding genes (Fig. 3). Further STRING database and RNA-seq analyses also support the positive association between BACH1 and glycolytic gene expression in lung cancer [9, 65]. Knockdown of BACH1 decreased the expression of HK2 and GAPDH to cancel out the rise of glucose metabolism-related indicators, and migration. Inhibitors such as 2-deoxyglucose (2-DG) and lonidamine (an HK2 inhibitor) block the glycolysis pathway at hexokinase, while 3-bromopyruvate (3-BP) represses GAPDH. Combined treatment with these drugs decreases glycolysis rates and effectively reverses the BACH1-induced migration of lung cancer cells. Moreover, BACH1 expression seems to be positively associated with PFKFB3 and SLC16A1 expression according to an analysis of human lung cancer datasets [9].

In addition, BACH1 is regulated by other signals and thus also indirectly influences glycolysis in cancer cells. It is reported that overexpression of BACH1 stimulated by long non-coding RNAs (lncRNA) small nucleolar RNA host gene 5 (SNHG5) regulates the levels of proteins related to glucose metabolism by targeting miR-299, thus regulating glucose consumption and lactate secretion, thereby facilitating the growth and glycolysis of breast cancer cells [66]. Furthermore, the induction of BACH1 deubiquitination by USP47 promotes the Warburg effect and progression in non-small cell lung cancer, as demonstrated by metabolism-related indicators [67]. Similarly, the reduction of UBR7 levels mediated by methyltransferase ALKBH5 reduces the transcription of Keap1, which in turn upregulates the Nrf2/BACH1/HK2 pathway, promoting tumorigenesis in hepatocellular carcinoma by targeting glycolysis [68]. In addition, BACH1, stabilized by the regulator of chromatin condensation (RCC2) at the C-terminal, induces the upregulation of HK2, which drives glioma progression [69].

#### Lactate metabolism

Lactate derived from aerobic glycolysis, which is an energy substrate for aerobic cancer cells and glucose-deprived immune cells, facilitates tumor invasion and metastasis, making it a significant prognostic factor in cancer patients [70–72]. BACH1 inhibits the expression of monocarboxylate transporter 1 (MCT1, encoded by SLC16A) and lactate dehydrogenase B (LDHB), downregulating lactate utilization in triple-negative breast cancer (TNBC) cells (Fig. 3) [73]. MCTs are the first step in the cellular catabolism of extracellular lactate [74, 75]. BACH1 deficiency enhances the lactate utilization of TNBC cells and increases their sensitivity to MCT1 inhibitors. Secondly, intracellular lactate is transported into mitochondria by MCT2 and MCT3 or transformed into pyruvate by LDHB, while the depletion of BACH1 enhances LDHB activity [73]. Thus, BACH1 regulates lactate efflux or influx, presenting promising targets for cancer treatment. In addition, BACH1 promotes the secretion of lactic acid, which was reported to potentially serve as a nutrient for regulatory T cells, thereby promoting immunotherapy resistance [10, 76]. However, further study of concrete mechanisms may reveal novel targets for immunotherapy.

### Tumor invasion and metastasis

**Fig. 4:**
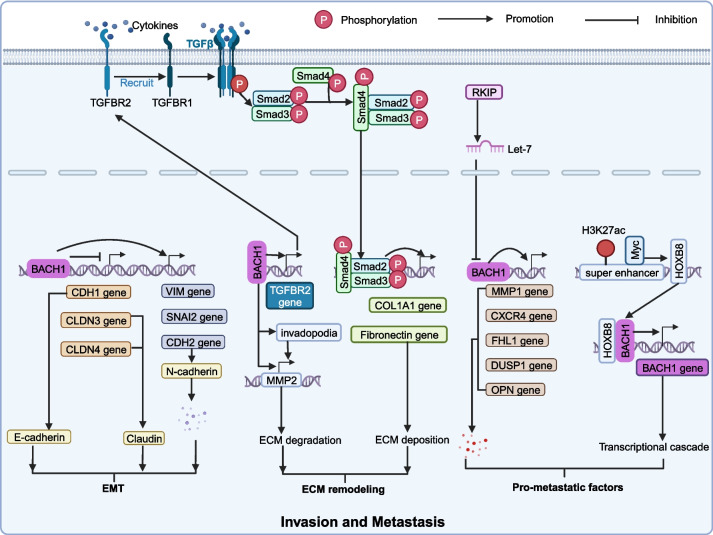
BACH1 promotes tumor invasion and metastasis. BACH1 plays a core regulatory role in the EMT, ECM remodeling, and expression of pro-metastatic factors thereby promoting tumor invasion and metastasis

Tumor invasion and metastasis include two stages: the dissemination of tumor cells from the primary tumor to distant tissues and organs and the adaptation of disseminated tumor cells to the distant tissue microenvironment. BACH1 facilitates tumor invasion and metastasis by regulating the EMT, ECM remodeling, and pro-metastatic factors (Fig. 4). At the same time, BACH1 upregulates the malignant features of cancer stem cells (CSCs).

#### EMT

The epithelial-mesenchymal transition (EMT) is a critical step in the initiation of cancer metastasis by downregulating epithelial genes and upregulating mesenchymal genes, thereby enhancing the motility and invasiveness of tumor cells [77]. In pancreatic ductal adenocarcinoma (PDAC), BACH1 indirectly inhibits the expression of epithelial gene CDH1 by directly reducing FOXA1 expression and promoting SNAI2 expression [11]. In addition, BACH1 directly inhibits the expression of CLDN3 and CLDN4, which are essential for the adhesion of epithelial cells. Knockdown of BACH1 enhances the expression of plakophilin-2 (encoded by PKP2). At the same time, CHIP-seq demonstrated that PKP2 is the target gene of BACH1 [11]. However, the precise regulation for PKP2 by BACH1 was not studied further. It was reported that the overexpression of BACH1 induced by tank binding kinase 1 (TBK1) increases intracellular labile iron and decreases the expression of E-cadherin (encoded by CDH1), thereby promoting the metastasis of pancreatic cancer cells [78]. In renal cell carcinoma, overexpression of BACH1 downregulates E-cadherin to decrease cell adhesion and promote invasion [79]. In esophageal squamous cell carcinoma, BACH1 directly induces the expression of CDH2, SNAI2, and VIM to promote the EMT by binding to their promoter regions [80]. In ovarian cancer, BACH1 recruits HMGA2 to the promoter region of Snail, thereby promoting the EMT and motility of tumor cells [81, 82]. In glioma, overexpression of BACH1 enhances the expression of CDH2, SNAI2, and CD44 in U87 cells to promote the EMT [83]. In hepatocellular carcinoma (HCC), BACH1 upregulates the expression of cell motility-related genes, such as insulin-like growth factor 1 receptor (IGF1R) and protein tyrosine kinase 2 (PTK2), to facilitate the growth and metastasis of HCC. However, IGF2, the ligand of IGF1R, upregulates the expression of BACH1 via the IGFR/(ERK1/2)/EST1 axis, creating a positive feedback loop [12]. Additionally, the induction of BACH1 via the lncRNA TRG-AS1/ miR-4500 axis promotes the proliferation, migration, invasion, and EMT of HCC [84]. Thus, BACH1 plays a critical role in the EMT process of various cancers.

#### ECM remodeling

BACH1 modulates extracellular matrix (ECM) remodeling to facilitate glioma invasion [83]. Studies have demonstrated that high levels of collagens and fibronectin support the infiltration and invasion of glioma [85, 86]. BACH1 activates the transforming growth factor beta (TGF-β) signaling pathway to facilitate the expression of ECM components in tumors [83, 87]. Specifically, transforming growth factor beta receptor 2 (TGFBR2) is an initial regulator of the TGF-β signaling pathway. The combination of TGFBR2 and cytokines initiates the TGFBR1/ smad2/3 /smad4 axis, which promotes the transcription of fibrotic genes (collagens and fibronectin) [83, 87]. BACH1 enhances the expression of TGFBR2 and activates the smad2/3 signaling pathway. In addition, BACH1 induces the formation of invadopodia, thereby promoting the expression and secretion of MMP2, which can help degrade the ECM [83]. BACH1 regulates the degradation and deposition of the ECM, creating a favorable environment for tumor metastasis (Fig. 4). The regulation of ECM remodeling by BACH1 is a novel approach to cancer treatment, which merits further research.

#### Pro-metastatic factors

There is increasing evidence that aberrant expression of specific factors facilitates tumor progression and metastasis. MMP1 regulates the adhesion between tumor cells, promoting tumor invasion and metastasis [88]. High expression of CXCR4 is closely related to increased tumor metastasis, and CXCR4 is involved in multiple processes related to metastasis, including chemotaxis, colonization and proliferation [89]. Similarly, FHL1, DUSP1 and OPN are critical for tumor invasion and metastasis. BACH1 has been identified as a central regulator of these tumor-associated genes related to the promotion of metastasis (Fig. 4). In colorectal cancer, BACH1 facilitates the expression of CXCR4 as demonstrated by TCGA dataset analysis, western blotting, and immunohistochemistry [90]. In breast cancer, bioinformatic analysis indicated that BACH1 mediates four Bone Marrow Signature (BMS) genes, including MMP1, CXCR4, FHL1, and DUSP1 [91]. Specifically, BACH1 directly promotes the expression of MMP1 by binding to its promoter region to facilitate metastasis [91, 92]. MMP9 induces tumor metastasis in pre-metastatic sites and facilitates carcinogenesis [93–95]. Conversely, silencing of BACH1 decreases the expression of MMP9 [96]. Raf kinase inhibitory protein (RKIP) is a tumor metastasis inhibitor that mediates the growth and differentiation of various organisms and exhibits reduced expression in many solid tumors [97–99]. BACH1 is repressed by RKIP, which targets let-7, thereby inhibiting breast cancer bone metastasis by downregulating the expression of BMS genes, including MMP1, OPN, and CXCR4 [92]. Interestingly, Lee et al. reported that BACH1 downregulates the transcription of both RKIP and itself [100]. Furthermore, myc drives the transcription of oncogenic HOXB8 by binding to its super enhancers. HOXB8 occupies and activates the transcription of the BACH1 gene with BACH1 itself and interacts with BACH1 to cause a transcriptional cascade, thereby enhancing colorectal cancer invasion [101].

#### Cancer stem cells

Cancer stem cells **(**CSCs), which are involved in tumor progression and metastasis, have been discovered in many types of cancer, including lung, liver, and pancreatic cancer [102–105]. In lung cancer, BACH1 promotes the expression of CD44^+^, thereby inducing the proliferation and invasion of lung CSCs both in vivo and in vitro [106]. In addition, BACH1 activates MAPK signaling to promote the growth and stemness of CSCs [106]. Although the downstream of MAPK signaling pathway related to CD44^+^ remains unclear, it is known that multifunctional factors (OCT4, SOX2 and NANOG) mediate the biological activity of CSCs [107]. In human embryonic stem cells, BACH1 recruits three multifunctional factors, namely NANOG, SOX2 and OCT4, and induces their deubiquitylation by recruiting deubiquitinase USP7, thereby stabilizing the three factors [31]. Thus, it is possible that BACH1 upregulates CSC-like properties by stabilizing multifunctional transcription factors, which is expected to be further corroborated in the future.

### Proliferation

A hallmark of cancer is replicative immortality, which is determined by many factors. BACH1 plays an essential role in regulating cancer cell proliferation. Activating mutations of Ras family proto-oncogenes are frequently found in human cancers. On the one hand, BACH1 inhibits HO-1 expression to maintain H-Ras^V12^-induced ROS accumulation, which facilitates ERK signaling. On the other hand, H-Ras^V12^ induces ERK signaling, which in turn suppresses the induction through negative feedback. As a resistor, BACH1 inhibits the feedback loop of ERK signaling for H-Ras^V12^. Taken together, BACH1 effectively facilitates H-Ras^V12^-induced ERK signaling, thereby promoting tumorigenesis and proliferation in the Ras-induced transformation MEFs model as a target of non-oncogene addiction by H-Ras^V12^ [108]. In PDAC, a lower level of BACH1 upregulates HO-1, activates AKT, ERK and eNOS, while also promoting the expression of HIF1A and VEGF as well as downregulating PTEN, thereby enhancing the proliferation of PDAC cells [109]. In Ewing sarcoma, BACH1 downregulates EWSR1, an RNA-binding protein that mediates the transcription percent of cell cycle protein D1a and D1b by raising the transcription elongation rate of Ewing sarcoma cells [6, 110]. Notably, circRNAs, lncRNAs and miRNAs act as upstream regulators of BACH1 in tumor cell proliferation. In osteosarcoma, BACH1 is induced by circ_0081001 via targeting miR-494-3p to promote proliferation of osteosarcoma cells [111]. Similarly, the circ_0000337/miR-4458/BACH1 axis also promotes proliferation in osteosarcoma [112]. In colorectal cancer, BACH1 is upregulated by circ_0087862 via sponging miR-142-3p to facilitate proliferation of colorectal cancer cells [113]. In hepatocellular carcinoma, BACH1 is stimulated by lncRNA712 via sponging miR-142-3p, thereby boosting proliferation [114]. In esophageal cancer, BACH1 is enhanced by lncRNA SNHG8 via downregulation of miR-1270 to promote proliferation [115].

Intriguingly, the function of BACH1 is different during tumor formation. Sato et al. found that BACH1 does not influence the proliferation of AsPC-1 cells when they are seeded at high concentrations, while inhibiting it at lower initial seeding concentrations. Moreover, these results have been verified in other cells, such as pancreatic ductal adenocarcinoma cell lines, SW1990 and BxPC-3 [11]. However, the specific mechanism remains unknown. In addition, the function of BACH1 is also different among various cell lines. Han et al. revealed that the expression of BACH1 is positively correlated with the increasing proliferation of the EOC cell line. At the same time, lower expression of BACH1 is also correlated with increasing proliferation of human umbilical vein endothelial cells [116]. Thus, BACH1 plays different roles in multiple cell types under specific experimental conditions, in line with its dual roles as either a transcriptional activator or inhibitor.

### Different cell death pathways influenced by BACH1

#### Ferroptosis

**Fig. 5:**
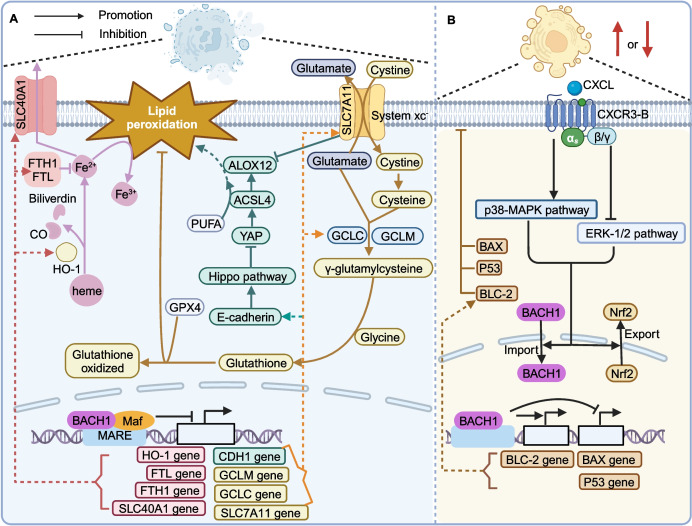
BACH1 plays an essential regulatory role in different cell death pathways. **A** The regulatory role of BACH1 in ferroptosis. **B** BACH1 exerts divergent effects on apoptosis in various cancers

Ferroptosis is a programmed cell death pathway related to necrosis. Ferroptotic cells form lipid peroxides and lipid hydroxyl radicals due to labile ferrous iron (Fe^2+^), resulting in cell death. BACH1 represses the expression of gene groups involved in L-glutathione (GSH) synthesis and labile iron metabolism to promote ferroptosis (Fig. 5) [117]. Firstly, in the ferritin-ferroportin pathway, BACH1 suppresses the expression of FTL (encoding ferritin light chain) and FTH1 (encoding ferritin heavy chain 1) to promote the redox activity of Fe^2+^. Furthermore, BACH1 suppresses the expression of SLC40A1 (encoding ferroportin, which transports Fe^2+^ out of the cytoplasm), thereby increasing the cellular concentration of labile iron and facilitating ferroptosis [117–122]. Interestingly, BACH1 reduces HO-1 activation and represses the accumulation of Fe^2+^, thereby protecting against ferroptosis. However, although HO-1 is upregulated by lower BACH1 levels, enough ferritin isolates Fe^2+^ generated by heme degradation to rescue cells from ferroptosis. Thus, BACH1-mediated HO-1 upregulation may repress or promote ferroptosis, which depends on the balance of HO-1 and ferritin [4, 123]. Secondly, in the GSH-GPX4 pathway, BACH1 suppresses the expression of GCLC and GCLM genes to inhibit the key enzyme of the GSH synthetic pathway, thereby promoting ferroptosis. Furthermore, BACH1 suppresses the expression of SLC7A11 to downregulate the subunit of system Xc- and inhibit the activity of arachidonic acid 12-lipoxygenase (ALOX12), which promotes lipid peroxidation and ferroptosis as a lipoxygenase [6, 117, 124, 125]. Notably, hotspot mutant p53^R175H^ abolishes BACH1-mediated downregulation of SLC7A11 thereby inhibiting ferroptosis to facilitate tumor growth. In addition, BACH1 expression induced by p53^R175H^ upregulates CEMIP (encoding cell migration inducing hyaluronidase 1) to promote tumor metastasis. The bidirectional role of BACH1 in cancer is mediated by p53^R175H^ by forming a ternary complex that regulates transcription [30]. Thirdly, in the Hippo pathway, BACH1 inhibits the expression of E-cadherin, which results in reduced intercellular contacts and deactivates the NF2-Hippo pathway to activate yes-associated protein (YAP, a ferroptosis-inducing transcriptional coregulator), thus promoting ferroptosis [126]. Fourthly, BACH1 may interact with Nrf2 and mediate the FSP1-CoQ pathway and tetrahydrobiopterin (BH4) system to promote ferroptosis, which is expected to be studied further [126].

Studies have proved that Stearoyl-CoA desaturase-1 (SCD1) protects tumor cells from ferroptosis in lung, gastric, and ovarian cancer [127–129]. Significantly, BACH1 inhibits the transcription of SCD1 to reduce oleic acid biosynthesis, thereby decreasing ferroptosis resistance [130–132]. Thus, BACH1 leads to a concentration gradient between cell membranes with low oleic acid content and lymph fluid with high oleic acid, resulting in the chemoattraction of cancer cells and metastasis via lymph vessels. To sum up, BACH1-induced ferroptosis advances lymph node metastasis but represses subcutaneous growth and hematogenous metastasis [132].

#### Apoptosis

BACH1 has different functions in the regulation of apoptosis in the progression of cancer (Fig. 5). BACH1 is highly expressed in lung CSCs, where it increases the expression of BCL-2 protein and represses the expression of BAX as well as p53, thereby inhibiting apoptosis [106]. However, in breast cancer, C-X-C motif chemokine receptor 3-B (CXCR3-B) induced by its ligands facilitates the activation of p38-MAPK and inhibits ERK-1/2, while also increasing the nuclear localization of BACH1 and nuclear export of Nrf2, leading to inhibition of CXCR3-B-associated signaling pathways, promoting apoptosis while inhibiting proliferation [133].

### Drug resistance in cancer

Drug resistance continues to be a persistent obstacle in cancer treatment. However, the mechanisms underlying chemotherapy resistance are still incompletely understood. BACH1 has been shown to confer resistance to temozolomide (TMZ) in glioblastoma. Specifically, BACH1 breaks the relationship between SP1 and p53 by competitively binding to p53 to abrogate the binding of SP1 to the promoter region of O-6-methylguanine-DNA methyltransferase (MGMT), thereby inducing MGMT expression and TMZ resistance. Thus, inhibitors targeting BACH1 may be a potential means of overcoming TMZ resistance [134]. In mantle cell lymphoma, BACH1 is stabilized by antioxidants blocking ROS, thereby aggravating bortezomib resistance instead of promoting cell death [135]. However, the downstream mechanism remains elusive. In breast cancer, BACH1 results in drug resistance related to mitochondrial metabolism. Metformin was reported to act as a potential antitumor drug by repressing the mitochondrial ETC complex I and other metabolic targets [136, 137]. Higher levels of BACH1 result in metformin resistance, and the effect is independent of pyruvate [10]. However, in shBACH1 cells, metformin resistance is only observed in the presence of pyruvate, which suggests that pyruvate levels affect metformin resistance by mediating the NAD^+^/NADH ratio. Furthermore, in BACH1-deficient cells, metformin, rotenone and antimycin A significantly repress cell growth and viability, meaning that BACH1 deficiency can effectively overcome cancer resistance to these drugs. Additionally, in BACH1-deficient cells, the induction of mitochondrial ETC genes such as COX15 or UQCRC1 commonly inhibits metformin resistance and cell growth. Notably, heme serves as an alternative trigger of BACH1 protein degradation, and heme administration emulates the knockdown of the BACH1 gene. In heme-treated TNBC cells, the sensitivity of metformin, rotenone and antimycin A increased significantly [10]. Thus, targeted inhibition of BACH1, as a promising strategy, increases the sensitivity to chemotherapy drugs that suppress mitochondrial metabolism in breast cancer and potentially other cancers [10]. Overall, BACH1 is involved in therapeutic resistance, and its downregulation provides a potential way to overcome drug resistance.

### Regulation of the tumor immune microenvironment

BACH1 is widely expressed in dendritic cells, neutrophils, monocytes, and macrophages that are abundant in the tumor microenvironment (TME). It is known that these immune cells are involved in nearly all aspects of tumor biology, including proliferation, differentiation, and metastasis. Hence, targeting BACH1 regulation in the TME is considered as highly promising anti-tumor therapeutic strategy [138]. BACH1 expression in immune cells affects tumorigenesis and cancer cells produce high ROS levels. In alveolar macrophages (AMs), ROS-activated BACH1 downregulates the expression of PDLIM2 [139]. The downregulation of PDLIM2 inhibits the phagocytosis of AMs, activates STAT3, and facilitates the tumorigenic polarization/activation of AMs. At the same time, the downregulation of PDLIM2 facilitates the pulmonary recruitment of monocytes and their differentiation into AMs, thereby suppressing the effect of cytotoxic T lymphocytes (CTLs). Taken together, BACH1 represses the innate and adaptive immune response in lung oncogenesis, thus playing a cancer-promoting role.

**Fig. 6:**
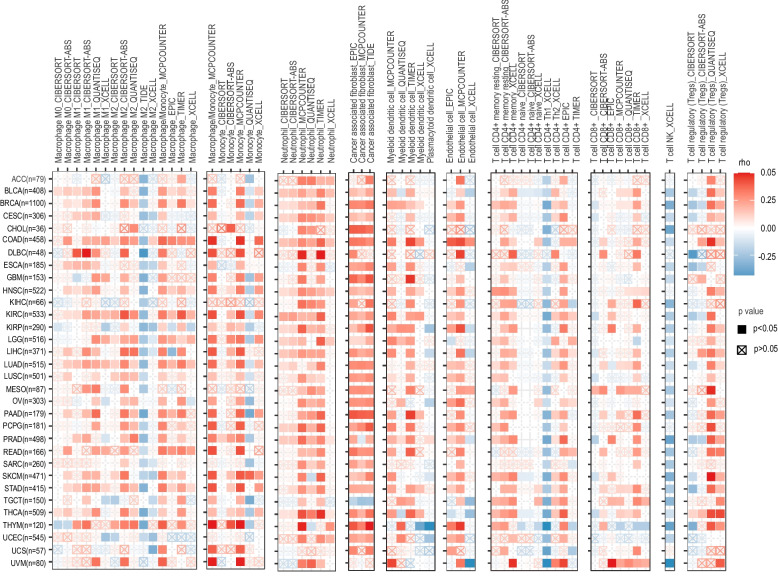
Analysis of the correlation between BACH1 and tumor-infiltrating immune cells in various cancers. Correlation between BACH1 and the abundance of multiple infiltrating immune cells in various cancers using TIMER 2.0 database. The purity-corrected partial Spearman’s rho values are displayed with statistical significance (*p*<0.05) marked with solid squares. ACC: Adrenocortical Carcinoma; BLCA: Bladder Cancer; BRCA: Breast Cancer; CESC: Cervical Cancer; CHOL: Cholangiocarcinoma; COAD: Colon Adenocarcinoma; DLBC: Large B-cell Lymphoma; ESCA: Esophageal Cancer; GBM: Glioblastoma; HNSC: Head and Neck Cancer; KICH: Kidney Chromophobe; KIRC: Kidney Clear Cell Carcinoma; KIRP: Kidney Papillary Renal Cell Carcinoma; LGG: Lower Grade Glioma; LIHC: Hepatocellular Carcinoma; LUAD: Lung Adenocarcinoma; LUSC: Lung Squamous Cell Carcinoma; MESO: Mesothelioma; OV: Ovarian Cancer; PAAD: Pancreatic Adenocarcinoma; PCPG: Pheochromocytoma and Paraganglioma; PRAD: Prostate Adenocarcinoma; READ: Rectal Adenocarcinoma; SARC: Sarcoma; SKCM: Skin Cutaneous Melanoma; STAD: Stomach Adenocarcinoma; TGCT: Testicular Cancer; THCA: Thyroid Carcinoma; THYM: Thymoma; UCEC: Endometrioid Cancer; UCS: Uterine Carcinosarcoma; UVM: Ocular melanoma

**Fig. 7:**
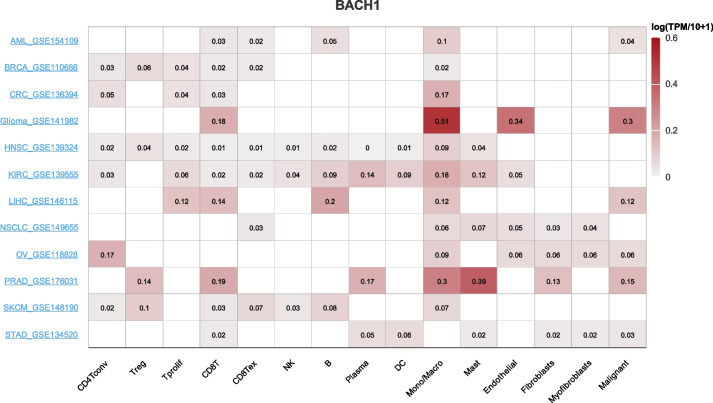
Correlation between BACH1 and TME at the single-cell level in the TISCH database. Heatmap showing BACH1 expression in various cells from diverse datasets. AML: Acute Myeloid Leukemia; BRCA: Breast Cancer; CRC: Colorectal Cancer; HNSC: Head and Neck Cancer; KIRC: Kidney Clear Cell Carcinoma; LIHC: Hepatocellular Carcinoma; NSCLC: Non-Small Cell Lung Carcinoma; OV: Ovarian Cancer; PRAD: Prostate Adenocarcinoma; SKCM: Skin Cutaneous Melanoma; STAD: Stomach Adenocarcinoma

Expression of BACH1 is related to tumor-infiltrating immune cells in different cancer types, and it is positively correlated with the level of tumor-infiltrating lymphocytes (TILs) in most cancer types according to the TIMER algorithm [76]. Further analysis indicated that BACH1-related genes are associated with ubiquitin-mediated proteolysis, T cell receptor, PD-1/PD-L1 expression, Th17 cell differentiation, endocytosis and other signaling pathways [76]. BACH1 is strongly associated with immune responses in glioblastoma, especially M0 and M2 tumor-associated macrophages (TAMs) [140]. Additionally, high BACH1 expression is positively correlated with increased expression of immune checkpoints (ICs, such as CD276, TIM-3, LAG3, TIGIT and LGALS9), TAM chemokines induced by glioblastoma cells (including MCP1, mGM-CSF, and EGF) and M2 TAM markers, which means that BACH1 contributes to an immunosuppressive TME in glioblastoma [140]. BACH1 is positively related to a high level of monocyte-myeloid-derived suppressor cells (Mo-MDSCs) in TNBC, which means that BACH1 may help cancer cells escape from immunosurveillance and repress immune overreaction, thus promoting tumorigenesis [141]. The specific mechanism is expected to be explored in vivo and in vitro in the future. In order to further understand the immunomodulatory role of BACH1 in various tumors, we deeply analyzed the correlation between BACH1 and various tumor-infiltrating immune cells using the TIMER 2.0 database (Fig. 6). In many tumors, BACH1 is positively correlated with multiple immune cells, especially macrophages, monocytes, neutrophils, cancer-associated fibroblasts, myeloid dendritic cells, and regulatory T cells. Among these immune cells, some play positive while others play negative roles in immunoregulation. Clarifying the implication of divergent effects contributes to our understanding of the roles of BACH1 in the tumor immune microenvironment. However, BACH1 is negatively associated with NK T cells, and it is worth investigating if it suppresses the NK-mediated killing of tumor cells. Moreover, we analyzed the correlation between BACH1 and TME at the single-cell level in the TISCH database. The results indicates that BACH1 is highly expressed in conventional CD4^+^ T cells, CD8^+^ T cells, B cells, monocyte-macrophages, and malignant cells in most tumors (Fig. 7). Notably, there was little difference between the results of TIMER 2.0 database analysis and single-cell level analysis in the TISCH database. The high dropout rate of present single-cell technologies leads to an abundance of incomplete gene tests. Overall, BACH1 has essential implications for the regulation of multiple immune cells. The roles of the significantly increased expression of BACH1 in various immune cells are yet to be explored, while the BACH1-mediated complex regulatory network in diverse cancer types and immune cells also merits further investigation.

## BACH1 as a therapeutic target

Based on its roles in benign and cancerous diseases, the blockade of BACH1 or its downstream effectors is a promising therapeutic strategy. In addition, some studies have demonstrated that their combination a contributes to the inhibition of tumor growth in cancer.

### Inhibiting BACH1

#### Small molecule inhibitors of BACH1

Some small molecule inhibitors of BACH1 are being investigated in benign diseases. As a non-psychotropic phytocannabinoid, cannabidiol induces the nuclear export and cytoplasmic proteasomal degradation of BACH1 in keratinocytes [142]. Isomeric O-methyl cannabidiol quinone (a novel cannabidiol derivative) not only has similar effects on BACH1, but also activates Nrf2 in cell-culture models of neurodegenerative diseases [143]. The non-electrophilic compound HPP-4382 inhibits the ability of BACH1 to bind DNA by imitating heme. However, HPP-4382 does not appear to affect the levels and nuclear distribution of BACH1 [144]. Similarly, HPP-D (an oral derivative of HPP-4382) competitively inhibits BACH1 from binding antioxidant response elements and promotes Nrf2 binding, thereby enhancing the expression of γ-globin in in sickle erythroid progenitors [145]. A substituted benzimidazole (HPPE) facilitates the release of BACH1 from DNA binding elements and nuclear export in a mouse model of Parkinson’s disease [65]. HPPE is an effective mimic of heme and has higher safety, which makes it a potential treatment for Parkinson’s disease. Based on virtual structural screening, the small molecule 1-piperazineethanol, α-[(1,3-benzodioxol-5-yloxy) methyl] -4-(2-methoxyphenyl) (M2) was identified as an inhibitor of BACH1 and effectively reduced aflatoxin B_1_-induced oxidative damage [146]. However, the research on these BACH1 inhibitors is still at the stage of in vitro experiments or animal models, while their bioavailability and safety need to be further explored. HPP971 from VTV-Therapeutics and ML-0207/ASP8731 from Astellas-Pharma are in clinical development as BACH1 inhibitors [147–149]. HPP971 has completed two phase I studies and was well tolerated.

As a potential antitumor strategy, small molecule inhibitors of BACH1 are also being explored in cancer. Intravenous hemin (Panhematin^®^) is approved for the treatment of acute porphyria by the Food and Drug Administration [150]. Hemin has no significant toxicity and decreases BACH1 levels [10, 20, 22]. Consistently, hemin treatment was found to enhance the sensitivity of cancer cells to MCT1 and mitochondrial inhibitors in tumor models [10, 73]. TBE53 (the biotinylated form of TBE31) has been demonstrated to effectively and persistently promote the degradation of BACH1 by the E3 ligase FBXO22 (50-fold more potent than hemin), while TBE31 has no influence on BACH1 levels. TBE53 (but not TBE31) reduces breast cancer cell migration and invasion in a BACH1-dependent manner [149]. Therefore, TBE53 is a potentially more potent alternative to hemin for inducing BACH1 degradation in anti-tumor therapy. However, its pharmacokinetic and pharmacodynamic properties needs further exploration in vivo. In addition, CDDO-trifluoromethyl-amide (CDDO-TFEA) and CDDO-Bardoxolone-Methyl (CDDO-Me) both decrease the nuclear levels of BACH1 to reduce invasion of lung cancer cells [151].

#### SiRNA

In addition to the small molecule inhibitors, siRNA-mediated gene therapy is a novel and effective antitumor strategy. In this approach, tumor cells are transfected with specific siRNAs that downregulate the expression of BACH1 in a dose-dependent manner, thereby repressing the migration and proliferation of breast cancer cells, leading to cell cycle arrest and promoting apoptosis [152]. BACH1 siRNA produces similar effects in prostate and HT-29 colon cancer cells, including inhibition of invasion and migration [96, 153].

### Inhibiting the downstream targets of BACH1

Antioxidants stabilize BACH1 to promote lung cancer metastasis, which depends on glycolysis and lactate secretion and is related to high ATP production rates. Small molecules such as 2-DG, lonidamine (a HK2 inhibitor), 3-BP (a GAPDH inhibitor), dichloroacetate (a pyruvate dehydrogenase kinase inhibitor that represses the production of lactate), and AZD3965 (MCT-1 inhibitor) repress antioxidant-induced cell migration in vitro. In addition, 3-BP and AZD3965 have a similar effect in vivo [9].

### Inhibiting both BACH1 and its downstream targets together

The genetic knockdown or pharmacological inhibition of BACH1 increases the use of lactate in TNBC cells, thereby sensitizing them to MCT1 inhibitors. Thus, combining hemin and MCT inhibitors (SR13800 and AZD3965) also presents a promising therapeutic strategy [73]. In addition, regulation of mitochondrial metabolism inhibits tumor growth by targeting BACH1, thereby sensitizing tumors to mitochondrial inhibitors. Hemin sensitizes TNBC to metformin by promoting the degradation of BACH1, making combined hemin-metformin treatment a potential therapeutic strategy for other cancer types, such as papillary thyroid carcinoma [10, 154]. Targeted inhibition of HO-1 promotes the FBXO22-dependent degradation of BACH1, thereby reducing cell migration and metastasis, which has been demonstrated in animal models. Treatment with zinc protoporphyrin IX (ZnPPIX) as a HO-1 inhibitor significantly suppresses the growth of gastric cancer, hepatocellular carcinoma, and sarcoma [155–158]. In addition, docosahexaenoic acid (DHA) -induced lipid peroxidation leads to the degradation of BACH1, promoting the expression of the HO-1 gene in human cancer cells [159]. Thus, combining DHA and HO-1 inhibitors represents a promising treatment for controlling tumor growth.

## BACH1 as a prognostic biomarker

Numerous studies have shown that BACH1 is a novel and promising prognostic biomarker. Pan-cancer analysis revealed that low BACH1 expression predicts better outcomes in patients with kidney papillary cell carcinoma, lower grade glioblastoma, hepatocellular carcinoma, pancreatic adenocarcinoma, sarcoma, and thyroid carcinoma. However, low BACH1 expression is associated with a worse prognosis in skin cutaneous melanoma [76]. The genetic variation rs372883C/T in the 3’-untranslated region of BACH1 is related to the risk of developing PDAC and gemcitabine sensitivity. The rs372883C variation leads to higher BACH1 levels than the rs372883T variation in normal pancreatic tissue and PDAC. Furthermore, patients with rs372883CC have a better response to gemcitabine and longer median survival time compared with rs372883TT patients [109]. Thus, the genetic variation rs372883C/T is a biomarker for gemcitabine treatment efficacy and prognosis. Lignitto et al. suggested that the levels and transcription characteristics of BACH1 are correlated with shorter survival, advanced clinical grade and stage, as well as metastasis in human lung adenocarcinoma [44]. BACH1 is a vital and independent predictor in early-stage lung adenocarcinoma. The expression level of BACH1 is negatively correlated with overall survival of patients [138]. In colorectal cancer, the high expression of BACH1 is positively correlated with a high clinicopathological stage [160]. The expression of BACH1 is markedly correlated with the pathological stage, tumor-node-metastasis (TNM) stage, distant metastasis, and survival outcomes. TNBC patients with higher BACH1 expression have shorter overall survival and disease-free survival, which suggests that BACH1 is a potential prognostic biomarker [161]. In addition, various polygenic risk models and compound nomogram models based on BACH1 have been established to assess the prognosis, such as the RKIP pathway metastasis signature (RPMS, for TNBC), BACH1 pathway metastasis signature (BPMS, an improvement from RPMS for TNBC), and a model for glioblastomas [140, 162].

## Perspectives

Past studies have shown that BACH1 promotes tumor progression in multiple ways and is a promising prognostic biomarker. Table 1 lists the oncogenic roles of BACH1 in different cancers. Furthermore, BACH1 regulates the transcription of diverse genes (Table 2). At the same time, BACH1 is also a downstream target as a core transcription factor (Table 3). Thus, BACH1-targeted regulation may be a novel strategy for antitumor therapy. Nevertheless, the mechanisms underlying BACH1 regulation and function still remain unclear in many cases. As mentioned earlier, given that BACH1 promotes ferroptosis, it is worth exploring the possibility of targeting BACH1 to facilitate ferroptosis in the tumor microenvironment. ROS play a vital role in the tumor microenvironment and affect various signaling pathways [163]. Thus, it is pertinent to explore whether BACH1 regulates these pathways due to its known effects on oxidative stress. As mentioned above, BACH1 exerts a significant impact on carcinogenesis, making it possible that a single targeted agent may be effective. However, recent studies indicated that BACH1 nonmonotonically mediates the expression of multiple genes, including RKIP, CXCR4, MMP1 and BACH1 itself, serving alternatively as a transcriptional activator or a transcriptional repressor at different protein levels [164]. At the same time, some previous studies revealed its cancer-suppressing effect, and BACH1 may negatively affect cancer cell survival by mediating protein homeostasis [165–167]. Cancer cells preferentially use the ubiquitin-proteasome system. The FBXO22-induced downregulation of BACH1 promotes AML progression, which means that BACH1 has a negative effect on tumor growth in this context [168]. Hence, a simple suppression for BACH1 may have negative effects on tumor metastasis in some cases [164]. The nonmonotone fitness landscape of BACH1 improves the accuracy and efficiency of targeted drug delivery, thereby increasing clinical benefits, which is an exciting direction worth pursuing in the future. According to KEGG pathway enrichment analysis, BACH1-associated genes are involved in signaling pathways related to PD-1/PD-L1 expression, T cell receptor function, and Th17 cell differentiation [76]. These findings prompt the question of how BACH1 affects these signaling pathways in the tumor immune microenvironment. In addition, in silico analyses of many tumor types suggest that BACH1 has a significant association with immune checkpoints and the tumor immune environment. These mechanisms deserve to be explored further as they may provide novel therapeutic strategies for both benign diseases and cancers.

**Table 1:** The oncogenic roles of BACH1 in different types of cancer

**Cancer type**	**Expression**	**Effects**	**Ref.**
Breast cancer	High expression in the TIMER 2.0 and CPTAC databases	Promotes mitochondrial metabolism, aerobic glycolysis, lactate metabolism, metastasis, and apoptosis	[10, 66, 73, 76, 82, 91, 92]
Lung cancer	High expression in lung stem cells	Promotes glycolysis, metastasis, and tumorigenesis	[9, 22, 67, 106]
Hepatocellular carcinoma	High expression	Promotes glycolysis, growth and metastasis	[12, 68]
Glioma	High expression in TIMER 2.0	Promotes glycolysis, progression, invasion and metastasis	[69, 83, 87]
Pancreatic adenocarcinoma	Low expression	Promotes metastasis and proliferation	[78, 109]
Ovarian cancer	High expression	Promotes metastasis and angiogenesis	[7, 81]
Colorectal cancer	High expression	Promotes invasion, metastasis, and angiogenesis	[28, 29, 90, 101, 113, 160]
Esophageal squamous cancer	High expression in TIMER 2.0 and significantly higher in metastatic tumor tissues	Promotes metastasis, proliferation, and angiogenesis	[76, 80, 115]
Osteosarcoma	-	Promotes progression and migration	[112]
Skin cancer	High expression	Promotes invasion and metastasis	[28, 29]

**Table 2:** The regulatory effects of BACH1 on downstream target genes

**Target gene**	**Effect**	**Function**	**Ref.**
HO-1	Repression	Maintenance of heme homeostasis and regulation of oxidative stress	[4]
YAP	Promotion	Facilitates the expression of YAP by binding to its promotor, in turn promoting the transcription of ICAM1 and VCAM1	[8]
PGC-1α	Repression	Reduces the production of ROS and improves angiogenesis after hindlimb ischemia	[51]
ANGPT-1	Repression	Inhibits angiogenesis	[33, 34, 51]
FGF-2			
VEGF			
IL-8			
Perp	Repression	Represses p53-induced cellular senescence	[35]
P21			
ITPR2	Repression	Decreases calcium efflux from the endoplasmic reticulum	[6]
CALM1	Repression	Activated when combined with calcium and represses the cell cycle	[6]
EWSR1	Repression	Mediates the transcriptional percent of cell cycle proteins D1a and D1b	[110]
Cebpb	Repression	Inhibits myeloid differentiation	[56]
Ebf1	Promotion	Promotes the differentiation of pro-B cells	[56]
Spic	Repression	Inhibits monocyte differentiation into red marrow and bone marrow macrophages	[59]
FIZZ1	Repression	Inhibits the differentiation of M2 macrophages	[61]
CD206			
YM1			
iNOS	Promotion	BACH1 deficiency in macrophages promotes NLRP3 inflammasome activation	[62]
COX-2	Repression		
HK2	Promotion	Supports aerobic glycolysis	[9]
GAPDH			
SLC16A	Repression	Reduces lactate utilization in TNBC cells to repress tumorigenesis	[73]
LDHB			
FOXA1	Repression	Promotes EMT-mediated tumor metastasis by inhibiting epithelial genes	[11, 78, 80, 81]
CLDN3			
CLDN4			
VIM	Promotion	Promotes EMT-mediated metastasis by inhibiting mesenchymal genes	
CDH2			
SNAI2			
IGF1R	Promotion	Promotes tumor growth and metastasis by upregulating the expression of cell motility-related genes	[12]
PTK2			
COL1A1	Promotion	Facilitates ECM remodeling to create an environment favorable to tumor metastasis	[83, 85, 86]
Fibronectin			
TGFBR2	Promotion	Activates the smad2/3 signaling pathway thereby promoting ECM remodeling	[87]
MMP1	Promotion	Promotes metastasis	[90–92]
CXCR4			
OPN			
FHL1			
MMP9	Promotion	Promotes angiogenesis and tumor metastasis	[82, 93, 94]
CEMIP	Promotion	Promotes metastasis	[30]
CD44^+^	Promotion	Induces stemness, proliferation and invasion of lung CSCs both in vitro and in vivo	[106]
SOX2	Promotion	Maintains CSC identity and promotes self-renewal	[106]
NANOG			
OCT4			
PDLIM2	Repression	ROS/BACH1/PDLIM2/STAT3 signaling pathway promotes lung tumorigenesis by driving alveolar macrophages	[139]
GCLC	Repression	Reduces oxidation and promotes ferroptosis	[6, 117, 121, 122, 124]
GCLM			
SLC7A11			
HIF1A	Repression	Inhibits the proliferation of PDAC	[109]
VEGF			
PTEN	Promotion		
FTH1	Repression	Affects iron homeostasis and promotes ferroptosis via the ferritin-ferroportin pathway	[117, 121, 122]
FTL			
SLC40A1			
CDH1	Repression	Promotes ferroptosis by suppressing the NF2-Hippo pathway and facilitates metastasis by promoting the EMT	[126]
VEGFC	Promotion	Promotes angiogenesis and tumor progression	[7, 80, 90]
MGMT	Promotion	Competitively binds to p53 and reduces its impact on MGMT expression	[134]

**Table 3:** The effects of upstream regulators on BACH1.

**Upstream regulator**	**Effect on BACH1**	**Function**	**Ref.**
SNHG5/miR-299	Upregulation	Upregulates BACH1, thereby regulating the levels of glucometabolic enzymes, glucose consumption and lactate secretion, facilitating proliferation	[66]
USP47	Stabilization	Inhibits the ubiquitination of BACH1	[67]
UBR7/Keap1/Nrf2	Downregulation	Induces the transcription of Keap1, thereby upregulating the Nrf2/BACH1/HK2 pathway	[68]
RCC2	Stabilization	Stabilizes BACH1 at the C terminal	[69]
Circ-0087862/ miR-142-3p	Upregulation	Promotes cellular proliferation, tumor invasion and migration	[113]
Circ-0000337/ miR-4458			[112]
SNHG8/ miR-1270			[115]
CXCR3-B/p38 MAPK&ERK-1/2	Stabilization	Increases the nuclear localization of BACH1	[133]
TBK1	Upregulation	Increases ferroptosis, thereby promoting metastasis	[78]
IGF/ IGFR/ERK1/2/EST1	Upregulation	Promotes tumor growth and metastasis by upregulating the expression of cell motility-related genes	[12]
RKIP/let-7	Downregulation	Inhibits metastasis	[97, 100, 101]
Myc/HOXB8	Upregulation	Activates transcription of BACH1 and enhances tumor invasion	[101]
p53^R175H^	Upregulation	Exerts a bidirectional effect on BACH1 in transcriptional regulation	[30]
miR-148a	Downregulation	Suppresses the maturation and homeostasis of B lymphocytes	[56, 57]

## Conclusions

This review systematically summarized the structure, regulatory mechanisms, and oncological significance of BACH1. However, the current research status provides little information on how BACH1 is post-translationally modified and dysregulated in cancer. In addition, the bioavailability and safety of BACH1 inhibitors remains to be investigated in clinical trials. Notably, BACH1 is highly expressed in almost all cancer types and plays a crucial role in the key processes of tumorigenesis including cancer metabolism, metastasis, proliferation, and drug resistance. Multiple reports illustrate that high BACH1 expression is associated with a poor prognosis. Thus, the crosstalk between BACH1 and other factors in the TME is a particularly fascinating area for future study. Overall, clarifying the mechanistic complexity of BACH1 will facilitate a deeper understanding of tumors on a fundamental level, as well as offering new avenues for translation from the lab bench into clinical applications, potentially leading to novel breakthroughs in targeted therapy.

## Data Availability

The datasets generated and/or analyzed during the current study are available in the UCSC database (https://xenabrowser.net/) and GDC database (https://portal.gdc.cancer.gov/).

## Abbreviations

2-DG: 2-deoxyglucose
3-BP: 3-bromopyruvate
AML: Acute myeloid leukemia
AMs: Alveolar macrophages
BACH1: BTB domain and CNC homology 1
BMS: Bone marrow signature
BRAF (V600E): B-Raf proto-oncogene variant
BTB: Broad-complex, Tramtrack, and Brab-a-brac
CIMP: A CpG island methylator phenotype
CLPs: Common lymphoid progenitors
CLS: Cytoplasmic localization signal
CNC-bZIP: cap ‘n’ collar and basic region leucine zipper
CP: Cysteine–proline
CREs: cAMP response elements
CRM1: Chromosome maintenance 1
CSCs: Cancer stem cells
CXCR3-B: C-X-C motif chemokine receptor 3-B
DHA: Docosahexaenoic acid
Ebf1: Early B-cell factor 1
ECM: Extracellular matrix
EMT: Epithelial-mesenchymal transition
ERK: Extracellular regulated protein kinases
ETC: Electron transport chains
GAPDH: Glyceraldehyde-3-phosphate dehydrogenase
GSH: L-glutathione
HCC: Hepatocellular carcinoma
HDAC1: Histone deacetylase 1
HK2: Hexokinase2
HO-1: Heme oxygenase-1
IGF1R: Insulin-like growth factor 1 receptor
Keap1: Kelch like ECH associated protein 1
LDHB: Lactate dehydrogenase B
lncRNA: Long non-coding RNA
MAREs: Maf recognition elements
MCT1: Monocarboxylate transporter 1
MEFs: Mouse embryonic fibroblasts
MGMT: O-6-methylguanine-DNA methyltransferase
Nrf2: Nuclear factor erythroid 2-related factor 2
PDAC: Pancreatic ductal adenocarcinoma
PDH: Pyruvate dehydrogenase
PDK: Pyruvate dehydrogenase kinase
RKIP: Raf kinase inhibitory protein
ROS: Reactive oxygen species
RPMS: RKIP pathway metastasis signature
SCD1: Stearoyl-CoA desaturase-1
TAM: Tumor-associated macrophage
TCA: Tricarboxylic acid
TCF4: Transcription factor 4
TGFBR2: Transforming growth factor beta receptor 2
TGF-β: Transforming growth factor beta
TME: Tumor microenvironment
TMZ: Temozolomide
TNBC: Triple-negative breast cancer
TREs: TPA response elements
VEGF: Vascular endothelial growth factor
